# Characteristics of Primary Dentition Occlusion in Preschool Children: An Epidemiological Study

**DOI:** 10.5005/jp-journals-10005-1143

**Published:** 2012-08-08

**Authors:** Sham S Bhat, HT Ajay Rao, K Sundeep Hegde, BS Kiran Kumar

**Affiliations:** Vice Principal, Professor and Head, Department of Pedodontics and Preventive Dentistry, Yenepoya Dental College, Mangalore-18 Karnataka, India, e-mail: viceprincipalydc@yenepoya.edu.in; Reader, Department of Pedodontics and Preventive Dentistry, Yenepoya Dental College, Mangalore, Karnataka, India; Professor, Department of Pedodontics and Preventive Dentistry Yenepoya Dental College, Mangalore, Karnataka, India; Reader, Department of Pedodontics and Preventive Dentistry, Century International Institute of Dental Sciences and Research Center Kasaragod, Kerala, India

**Keywords:** Primary dentition, Occlusion, Molar relationship, Canine relationship, Open bite, Crossbite, Infraocclusion, Scissors bite

## Abstract

**Objective:** The establishment and maintenance of normal occlusion constitutes one of the important objectives of pediatric dentistry. There are very few studies assessing the occlusal characteristics of primary dentition in the preschool children. The objective of this study was to assess the occlusal characteristics of primary dentition and difference in various parameters, in children between 3 and 5 years of age.

**Materials and methods:** Eight hundred and eight healthy children, of age group between 3 and 5 years, and having full set of deciduous dentition, were selected by random sampling. Examination and recording of occlusal characteristics were done by single examiner.

**Results:** Flush terminal molar relationship was seen in 67.9% of children. Statistically significant (χ^2^ = 47.835, p = 0.001) increase in mesial step molar relationship was seen with age. The class I canine relationship was the most prevalent canine relation, however the agewise changes of canine relation were not statistically significant. Anterior open bite was observed in 0.2% of children, which was less than the prevalence reported earlier in literature. 91.2% of children had overjet of 1 to 2 mm. The incidence of anterior and posterior crossbite was 0.4%. No cases of infraocclusion and scissors bite were observed.

**Conclusion:** There is change in deciduous molar relationship as age increases. There is a significant increase in mesial step molar relation in 5 years age group compared to 3 years. The low prevalence of posterior crossbite and anterior open bite is suggestive of lower prevalence of sucking habits in children. Larger sample size may be required to assess the prevalence of infraocclusion and scissors bite.

**How to cite this article:** Bhat SS, Rao HTA, Hegde KS, Kumar BSK. Characteristics of Primary Dentition Occlusion in Preschool Children: An Epidemiological Study. Int J Clin Pediatr Dent 2012;5(2):93-97.

## INTRODUCTION

There is little information on the changes of the occlusal pattern during the period of deciduous dentition. The prevalence of all malocclusions in the primary dentition is also not thoroughly reported.^1^ Analysis of the occlusion in the primary dentition should consider the arrangement of deciduous teeth and the occlusal relationship of the anterior and posterior segments of both arches. The recognition of normal occlusion patterns in primary dentition as well as the identification of morphologic changes during permanent teeth eruption is essential for treatment planning in pediatric dentistry.^2^

It is agreed that in the deciduous dentition it is common to have spacing between the teeth and for the second molars to have a flush terminal plane relationship.^3,4^ At the time of eruption of the first permanent molar, their initial occlusion is dependent on the terminal plane relationship of the deciduous second molars.^2^

The prevalence of flush terminal plane decreases with age, whereas the mesial step demonstrates a corresponding increase in frequency. Therefore, around 6 years of age, the mesial step predominates at flush terminal plane, providing a favorable molar relationship in the primary dentition for a direct intercuspation of the erupting permanent molars. As skeletal growth pattern overcomes any dental adjustment mechanisms, a distal step in the primary dentition probably reflects a skeletal imbalance, and typically results in a class II malocclusion in the permanent dentition.^5-9^ Class III malocclusion is much less common than class II, but a child who has a mesial step relationship at an early age is at some risk of developing class III malocclusion.^10^ The relationship between deciduous canines is also a reliable reference criterion for the assessment of the anteroposterior occlusion, especially if the second molars are lost.^11^

Bouge in 1908 stated that if malocclusions were found in the primary dentition, the same occlusal problems would be expected to occur in the succeeding permanent dentition.^12^ There has been an increased awareness of the role of deciduous dentition in the determination of permanent tooth position and occlusion. The data shows that the malocclusion varies greatly with the population studied and the method of notation. The wide variations in individuals depend on oral habits, attrition and normality are difficult to define.

In view of the limited number of studies pertaining to occlusion in preschool children and the contradictory findings concerning age changes in deciduous molar relation, a further investigation seemed appropriate. Therefore the objective of our study was to assess the occlusal characteristics of primary dentition in the age group of 3 to 5 years and agewise differences with various parameters including terminal molar relation, primary canine relation, overbite, overjet, anterior crossbite, posterior crossbite, infraocclusion and scissor bite.

## MATERIALS AND METHODS

Eight hundred and eight children (423 males and 385 females) of age group of 3 to 5 years, from all nursery schools in and around the city of Mangalore, India were selected. Stratified cluster random sampling method was adopted. The schools were randomly selected from the list. All children, irrespective of socioeconomic status whose age was between 3 and 5 years, had complete set of deciduous dentition and healthy were selected for the study from these selected schools. The age of the child was obtained from school records. Those children who had grossly decayed or any permanent teeth were excluded from the study. Occlusal assessment was done only on children who had complete primary dentition without any erupted permanent teeth and free from extensive caries that would affect the mesiodistal or occlusogingival dimension of a tooth and therefore influence the occlusal characteristics. Each child was examined while lying in supine position with his or her head resting on the examiner's lap (who sat on one of the chairs) and feet on another chair. One examiner with the aid of penlight, mouth mirror and a metal millimeter ruler performed examination throughout the study. Occlusion was assessed with the teeth in centric occlusion. The following arch characteristics were recorded by a single examiner throughout the study using published definitions:^13^

1. ***Terminal plane relationship of the second primary molars:***
  - *Flush terminal:* The distal surfaces of the upper and lower second primary molars in the same vertical plane in centric occlusion.
  - *Distal step:* The distal surfaces of the lower primary second molar in posterior relationship to the distal surface of the upper second molars in centric occlusion.
  - *Mesial step:* The distal surfaces of the lower primary second molar in anterior relationship to the distal surface of the upper second molars in centric occlusion.
2. ***Primary canine relationship:***
  - *Class I:* The tip of the maxillary primary canine tooth is in the same vertical plane as the distal surface of the mandibular primary canine.
  - *Class II:* The tip of the maxillary primary canine tooth is mesial to the distal surface of the mandibular primary canine.
  - *Class III:* The tip of the maxillary primary canine is distal to the distal surface of the mandibular primary canine.
3. ***Degree of overbite:***It was graded according to coverage of the mandibular incisor by the most protruded fully erupted maxillary incisor.
  - *Normal:* Coverage of up to half of the mandibular incisor by the maxillary incisor.
  - *Increased:* Coverage of more than half of the mandibular incisor by the maxillary incisor.
  - *Edge-to-edge*
  - *Anterior open bite*: Negative overlap in the vertical plane.
4. ***Degree of overjet:***It was measured from the palatal surface of the mesial corner of the most protruded fully erupted maxillary incisor to the labial surface of the corresponding mandibular incisor. Degree of overjet was recorded in millimeters.
5. ***Anterior crossbite:***It was recorded when one or more of the maxillary incisors occluded lingual to the mandibular incisors.
6. ***Posterior crossbite*:** It was recorded when one or more of the maxillary primary canines or molars occluded lingual to the buccal cusps of the opposing mandibular teeth.
7. ***Infraocclusion:***It was recorded in the canine or molar segments if the distance between one or more fully erupted opposing teeth was at least 2 mm.
8. ***Scissors bite:***It was recorded when one or more maxillary primary molars occluded buccal to the buccal surfaces of the corresponding mandibular teeth.

**Table 1:** **Table 1:** Terminal molar relation

*Age*		*Flush terminal*				*Mesial step*				*Distal step*				*Total*	
		*Right*		*Left*		*Right*		*Left*		*Right*		*Left*			
3 years		311 (78.9%)		313 (79.4%)		78 (19.8%)		76 (19.3%)		5 (1.3%)		5 (1.3%)		394 (100%)	
4 years		167 (59.2%)		167 (59.2%)		105 (37.2%)		105 (37.2%)		10 (3.5%)		10 (3.5%)		282 (100%)	
5 years		69 (52.3%)		70 (53.0%)		60 (45.5%)		59 (44.7%)		3 (2.3%)		3 (2.3%)		132 (100%)	
Total		547 (67.7%)		550 (68.1%)		243 (30.1%)		240 (29.7%)		18 (2.2%)		18 (2.2%)		808 (100%)	

For right side: χ^2^ = 47.835, p = 0.001 (very highly significant); For left side: χ^2^ = 48.704, p = 0.001 (very highly significant)

## RESULTS

A total of 423 males and 385 females were examined. Of these 394 were 3 years of age, 282 were 4 years and 132 were 5 years of age. Males and females were pooled in each age group as there were no significant differences between them with respect to occlusion.

The distribution of different sagittal relationships of molars is shown in Table 1. Percentage distribution of terminal molar relationship showed flush terminal molar relation in 67.7% of subjects on right side and 68.1% on left side. The mesial step was seen in 30.0% on right side and 29.7% on left side. Distal step was seen in 2.2% in both right and left side. The agewise changes in terminal molar relationship were statistically very highly significant.

The percentage distribution of canine relation was class I in 88.9%, class II in 7.2% and class III in 4.0% in right side. On the left side the percentage distribution was class I in 89.0%, class II in 7.1% and class III in 4.0%. The change in agewise distribution of canine relation was not statistically significant (Table 2).

The degree of overbite when statistically analyzed, was observed that 67.2% had normal overbite, 31.7% had increased overbite, 0.9% had edge-to-edge bite and 0.2% had open bite. The agewise changes in degree of overbite were statistically insignificant (χ^2^ = 11.626, p = 0.071) (Table 3).

The overjet was measured in millimeters using a millimeter scale. The frequencies of overjet of one, two, three, four and five millimeter were 14.1, 77.1, 7.1, 1.6 and 0.1% respectively. Overall the mean overjet was 1.9653 with SD of 0.5351. The agewise changes in overjet values were not statistically significant (p = 0.641).

The anterior crossbite was seen in 0.4% of sample. Of the 394 children of age group of 3 years, only 0.3% had anterior crossbite, while no cases of crossbite was observed in age group of 4 years, and in age group of 5 years. 1.5% of cases of anterior crossbite were observed. The agewise changes were not statistically significant (χ^2^ = 5.87, p = 0.053).

Posterior crossbite was assessed for their presence or absence. 0.4% of children showed presence of unilateral posterior crossbite. In age group of 3 years, there were no cases of crossbite, while in age group of 4 and 5 years, there were 0.7 and 0.8% posterior crossbite respectively. The agewise changes in the incidence of posterior crossbite were however, not statistically significant (χ^2^ = 2.871, p = 0.238).

The presence of infraocclusion and scissors bite was assessed in the samples. However, no cases were seen in any age group.

**Table 2:** **Table 2:** Primary canine relation

*Age*		*Class I*				*Class II*				*Class III*				*Total*	
		*Right*		*Left*		*Right*		*Left*		*Right*		*Left*			
3 years		358 (90.0%)		359 (91.1%)		24 (6.1%)		23 (5.8%)		12 (3.0%)		12 (3.0%)		394 (100%)	
4 years		251 (89.0%)		251 (89.0%)		21 (7.4%)		21 (7.4%)		10 (3.5%)		10 (3.5%)		282 (100%)	
5 years		109 (82.6%)		109 (82.6%)		13 (9.8%)		13 (9.8%)		10 (7.6%)		10 (7.6%)		132 (100%)	
Total		718 (88.9%)		719 (89.0%)		58 (7.2%)		57 (7.1%)		32 (4.0%)		32 (4.0%)		808 (100%)	

For right side: χ^2^ = 8.064, p = 0.089 (not significant); For left side: χ^2^ = 8.071, p = 0.076 (not significant)

**Table 3:** **Table 3:** Degree of overbite

*Age*		*Normal*		*Increased*		*Edge-to-edge*		*Anterior open bite*		*Total*	
3 years		253 (64.2%)		137 (34.8%)		4 (1.0%)		0		394 (100%)	
4 years		201 (71.3%)		80 (28.4%)		0		1 (0.4%)		282 (100%)	
5 years		89 (67.4%)		39 (29.5%)		3 (2.3%)		1 (0.8%)		132 (100%)	
Total		543 (67.2%)		256 (31.7%)		7 (0.9%)		2 (0.2%)		808 (100%)	

χ^2^ = 11.626, p = 0.071 (not significant)

## DISCUSSION

It is important that conditions that predispose one to develop a malocclusion of the permanent dentition be detected early in the primary dentition. The understanding of anterior and posterior changes that occur in occlusion between primary and early permanent dentition is crucial for the clinician involved in planning early interceptive treatment.^14^

Our study done on Indian population showed a percentage distribution of flush terminal molar relation in 67.7% of subjects on right side and 68.1% on left, which is comparable to studies done by Alexander et al^1^ and Otuyemi et al on Nigerian population of same age group.^12^ Another study done on Indian population by Nanda et al^8^ who carried out study on 2 to 6 years age group found flush terminal plane in 72% of children. Findings observed in our study, though based on cross sectional investigations, showed an increase in the mesial step molar relationship with advancing age. These changes may be attributed to the forward growth of the mandible. Studies by Infante PF^15^ showed that there was decrease in distal step molar relationship as age increases. But studies by Nanda et al^8^ and Ravn JJ^16^ showed that it was invariably maintained throughout the primary dentition stage and was always transferred unchanged to the permanent dentition. However, in this study, we could not correlate the changes in the distal step molar relationship with age. Patients with a flush terminal relationship present a more challenging diagnostic question.

The prevalence of canine class II relationship in our study is 7.2%; much lower than 45% in English^17^ and the 31.6% reported by Ravn JJ in Danish^16^ children. The difference could be due to small sample size in the former study and inclusion of children with extracted teeth in latter study. In addition both the studies included children from young age group (3 years). It has been suggested that the prevalence of class II canine relationship seems to decrease with advancing age due to termination of some environmental factors such as sucking habits in the older age group, though not observed in the our study.

The prevalence of normal overbite was found in about 67.2% of population comparable with several studies.^12,18-20^ However, the prevalence of anterior open bite was 0.2% and was significantly less than that found in Western population. Otuyemi et al^12^ also showed that anterior open bite was seen in 5.3% of same age group in Nigerian children. No obvious reason could be offered to explain the difference except that oral habits, such as dummy sucking and finger sucking may play a significant role.^12^ This may be due to lesser prevalence of habits like thumb sucking in Indian population than in Western population.

The mean overjet was 1.9 mm. The prevalence of overjet above 2 mm was 8.8%, which was comparable to Nanda et al.^8^ However, it was much lower than the studies done by Foster TD^17^ and Infante PF.^15^ Nanda et al^8^ has shown that there was a significant reduction in overbite and overjet as children become older. The changes observed in this study however were not significant.

The prevalence of posterior crossbite in the present sample was 0.4%. This is lower than the earlier reported incidence of posterior crossbite by Infante PF^15^ and other studies done on North American and European population. It is observed that Caucasian population generally exhibited higher prevalence of posterior crossbite than African and Asian populations.^12,13,16^ The different prevalence of posterior crossbite between the different cultures may be due to the difference in prevalence of sucking habits. However, larger sample size may be required to assess the prevalence of posterior crossbite in Indian population. All cases observed in this study were of unilateral type, similar to other studies. The prevalence of anterior crossbite in the studied population is 0.4%. The prevalence of anterior crossbite in Saudi population was 1.7% and in English population 1.0%. However, it is lower than the prevalence of anterior crossbite in Finnish^18^ and African-Americans.^19^

Infraocclusion or submerged primary molar was not found in any of the present study. This occlusal anomaly is found in a frequency of only 0.6% in Nigerian population, 0.5% in Australian aborigines and no cases in West Indian patients.

However, a high prevalence of infraocclusion was found in studies done among Israeli children, that is prevalence of 24.8%, prevalence of 6.9% in North American Whites of Scandinavian ancestry, prevalence of 8.9% in Swedish children and 2.5% in British children.^20^ It is apparent that racial factors may have an important role in the development of submerged teeth.^12^

Scissors bite was not seen in any of the cases we observed. This occlusal anomaly is reported in very few epidemiological studies. No cases were observed in Saudi children in studies done by Farsi et al^13^ and only 14 cases were observed in study done by Kisling et al^21^ on 1396 Dutch children. Larger sample size may be necessary to assess the number of cases of scissors bite in Indian children.

## CONCLUSION

The present study is a cross-sectional study. It provides an insight into patterns of occlusal relationship and its changes with age in Indian preschool children. This study confirms the finding reported earlier that flush terminal molar relationship is the most prevalent terminal molar relationship in deciduous dentition. There is a statistically significant increase in mesial step molar relationship with age. The prevalence of anterior open bite in the studied population is comparatively less than that reported earlier. Lower prevalence of posterior crossbite and anterior open bite is suggestive of lower prevalence of abnormal sucking habits in Indian population. Further longitudinal studies are needed to assess the changes in occlusal pattern from the deciduous dentition to permanent dentition. Also larger sample is probably required to assess the incidence of cases of infraocclusion and scissors bite.
